# Outlier classification performance of risk adjustment methods when profiling multiple providers

**DOI:** 10.1186/s12874-018-0510-1

**Published:** 2018-06-15

**Authors:** Timo B. Brakenhoff, Kit C. B. Roes, Karel G. M. Moons, Rolf H. H. Groenwold

**Affiliations:** Julius Center for Health Sciences and Primary CareUniversity Medical Center Utrecht, PO Box 85500, Utrecht, 3508 GA the Netherlands

**Keywords:** Propensity score, Risk adjustment, Classification, Profiling, Random effects, Logistic regression, Simulation study

## Abstract

**Background:**

When profiling multiple health care providers, adjustment for case-mix is essential to accurately classify the quality of providers. Unfortunately, misclassification of provider performance is not uncommon and can have grave implications. Propensity score (PS) methods have been proposed as viable alternatives to conventional multivariable regression. The objective was to assess the outlier classification performance of risk adjustment methods when profiling multiple providers.

**Methods:**

In a simulation study based on empirical data, the classification performance of logistic regression (fixed and random effects), PS adjustment, and three PS weighting methods was evaluated when varying parameters such as the number of providers, the average incidence of the outcome, and the percentage of outliers. Traditional classification accuracy measures were considered, including sensitivity and specificity.

**Results:**

Fixed effects logistic regression consistently had the highest sensitivity and negative predictive value, yet a low specificity and positive predictive value. Of the random effects methods, PS adjustment and random effects logistic regression performed equally well or better than all the remaining PS methods for all classification accuracy measures across the studied scenarios.

**Conclusions:**

Of the evaluated PS methods, only PS adjustment can be considered a viable alternative to random effects logistic regression when profiling multiple providers in different scenarios.

## Background

In the last decades, performance of health care providers, for instance hospitals, has come under immense scrutiny. Government institutions, patients and providers themselves are increasingly demanding performance indicators of the quality of care. These can be based on clinical outcome measures such as mortality or complication rates [1–3]. For example, when profiling (i.e., assessing the performance of) well-established, high-risk procedures such as coronary artery bypass grafting (CABG), mortality is considered an appropriate outcome measure and thus often used [2–4]. After adjustment for differences in patient characteristics between providers, these mortality rates can be used to classify providers as performing as expected (*normal*) or either better or worse than expected (*outlying*). Unfortunately, when using customary methodologies to adjust these outcome measures across providers, misclassification of provider performance is not uncommon, which may in turn have immense economic and societal implications [5–8].

When making comparisons between health care providers, an essential step is the adjustment for differences between providers in the risk profiles of their patients. This is often referred to as risk adjustment. Taking into account the differences in relevant patient characteristics between providers (also known as case-mix) is crucial to obtain accurate and reliable estimates of provider performance [1, 9]. However, many studies have found that traditional regression based methods lead to inadequate adjustment for case-mix and are thus unable to correctly classify providers in a consistent manner. In addition, this classification performance is highly dependent on the statistical model applied and the classification criteria used [1, 3, 6, 10–13], especially when low-volume providers are included or outcomes are rare [14–17].

Propensity score (PS) methods have previously been put forward for risk adjustment [18]. These methods showed superior performance over conventional multivariable regression in several observational dichotomous treatment settings, e.g. when samples are small [19–27]. Furthermore, a simulation study [28] found that some PS methods performed on par with multivariable regression when profiling several providers, in line with results found in analogous settings where multiple treatment options were compared [29–32]. Seeing as PS methods have certain attractive advantages over conventional regression including the easy assessment of balance on relevant case-mix variables between multiple providers and their flexibility for different types of outcomes [20, 22], PS methods are considered viable alternatives for risk adjustment prior to provider profiling.

However, extended methodological research on the performance of PS and regression based methods when profiling many providers are still lacking [33]. The aim of this study was to compare several PS methods with conventionally used (hierarchical) logistic regression on their ability to identify (or classify) health care providers that performed better or worse than expected (i.e. outliers). A simulation study, based on empirical data from the field of cardiac surgery, was used to assess how the classification accuracy of each method differed in varying circumstances that may be encountered in practice.

## Methods

### Risk adjustment methods

Before detailing the set up of the simulation study, the following risk adjustment methods are explained: fixed effects logistic regression (*LR*_*F*_), random effects logistic regression (*LR*_*R*_), generalized propensity score (gPS) case-mix adjustment (*gPS*_*A*_), gPS inverse probability weighting (*gPS*_*W*_), gPS inverse probability weighting with trimming (*gPS*_*WT*_) and gPS marginal mean weighting through stratification (*gPS*_*MWS*_).

#### Fixed and random effects logistic regression

When dealing with dichotomous outcomes, such as mortality, multivariable logistic regression models are traditionally used for risk adjustment. These models can include the individual providers of which we want to determine the performance as either fixed or random effects. Fixed effects logistic regression (*LR*_*F*_) assumes that all variation between providers is due to differences in case-mix and that the model specification is correct. By including providers as dummy variables, direct comparisons between providers can be made [34, 35]. Random effects logistic regression (*LR*_*R*_) accounts for the increased similarity between patients attending the same provider, the hierarchical structure of the data, and allows for residual variance between providers that may not be attributable to performance. In addition, the dimensionality of the model is greatly reduced by only estimating the parameters of the distribution underlying the provider effects [36]. *LR*_*R*_ is considered especially suitable when between-provider variation is to be quantified, provider-level variables are measured, or low volume providers are to be profiled [6, 13, 34, 37, 38].

How the provider effects are included in the model can have profound consequences on the accuracy of classifying providers as either normal or outliers. As provider effects are assumed to come from an underlying distribution in *LR*_*R*_, effect estimates of providers (especially those with low volume) can borrow information from the other providers, shrinking these effects towards the mean of all providers [34]. This results in the identification of fewer performance outliers as compared to when *LR*_*F*_ is used [35–40]. Given the fundamental difference in how the model is formulated, the decision whether to use *LR*_*F*_ or *LR*_*R*_ is largely dependent on the goal of the profiling exercise. At present, most papers advocate the use of *LR*_*R*_ due to the hierarchical nature of provider profiling, and its conservativeness in identifying outliers.

#### Generalized propensity score methods

The propensity score (PS) was defined by Rosenbaum and Rubin in 1983 as “the conditional probability of assignment to a particular treatment given a vector of observed covariates” [25]. They demonstrated that in observational studies for causal effects, adjustment for PSs was sufficient to remove bias due to observed covariates assuming exchangeability and positivity (referred to as ignorability by Rosenbaum and Rubin [25]). Exchangeability requires that the conditional probability of receiving the treatment only depends on observed covariates and not on the outcome. Positivity implies that the probability of receiving any treatment given observed covariates is positive. For health care provider profiling, the received *treatment* is not a medical intervention but instead the *provider* attended. When comparing two providers, each patient’s PS is their fitted probability of attending one of the providers, estimated by regressing the provider indicator on the observed case-mix variables using a logistic regression model. Note that some *strong* predictors of provider attendance, such as the patient’s address, may be omitted from this model as they are not expected to be related to an outcome such as mortality and thus do not qualify as a confounder. For multiple provider comparisons, the generalized propensity score (gPS) can be used to adjust for observed case-mix variables. The gPS is described by Imbens [29] as the conditional probability of attending a particular provider given case-mix variables, and was further developed by Imai & van Dyk [41]. The gPSs of each patient for each provider can be estimated using multinomial logistic regression including all relevant observed case-mix variables.

There are several different ways to utilize the extracted gPSs to determine the average performance of each provider. In gPS case-mix adjustment (*gPS*_*A*_), provider effects on the outcome are conditional on the gPSs (for further details see: [31, 42]). For gPS weighting (*gPS*_*W*_) the sample is first re-weighted by the inverse gPS of the provider actually attended. In the weighted sample, marginal provider effects can be estimated by only including the providers in the outcome model (for further details see: [31]). Extreme weights can be trimmed to a certain percentile to reduce the influence of outlying weights and potential model misspecification (as applied in *gPS*_*WT*_). However, this can also lead to biased estimates due to inferior risk adjustment [43]. *gPS*_*MWS*_ combines elements of gPS stratification and *gPS*_*W*_ and has been suggested to be superior to *gPS*_*W*_ in both a binary and multiple treatment setting [32, 44, 45]. In this method, the gPSs for each provider are first stratified into several categories prior to weighting each individual by his/her representation within their stratum. Subsequently, marginal provider effects can be estimated just as in *gPS*_*W*_ (see [44] for a detailed description). While other methods have also been described in the literature, such as gPS stratification [46] or gPS matching [30, 46, 47], these methods have either been shown to perform worse than the aforementioned methods [22, 27, 48, 49] or are logistically impractical when dealing with large numbers of providers [30, 44, 47].

### Simulation study

A Monte Carlo simulation study was conducted based on empirical data from the field of cardiac surgery. This allowed us to mimic a situation with perfect risk adjustment in which the observed outlier classification accuracy of each method was compared with true outlier status as fixed in each generated dataset. Several parameters were varied across different scenarios each simulated 1000 times (see section *Scenarios*). Simulations were peformed using R (v3.1.2) [50]. R scripts used for the simulation study are available upon request.

#### Data source

Open heart surgery is a field that has been subject to many developments in risk-adjusted mortality models for quality control in the last decades [4, 40]. A selection of anonymized data from the Adult Cardiac Surgery Database provided by the Netherlands Association of Cardio-Thoracic Surgery was used as a realistic foundation for the simulation study.

The Adult Cardiac Surgery Database contains patient- and intervention characteristics of all cardiac surgery performed in 16 centers in the Netherlands as of 1 January, 2007. This dataset has previously been described and used by Siregar et al. for benchmarking [51, 52]. For the simulation study described in this paper, all patients from the 16 anonymized centers undergoing isolated CABG with an intervention date between 1 January, 2007 and 31 December, 2009 were included in the cohort. The average in-hospital mortality was 1.4%, ranging from 0.7 to 2.3%. The center indicator variable and outcome measure (in-hospital mortality) were removed from the dataset. Of the dichotomous variables included in the EuroSCORE, only those with an overall incidence over 5% were used. The final dataset was thus comprised of the following eight relevant predictors of mortality following CABG: age (centered), sex, chronic pulmonary disease, extracardiac arteriopathy, unstable angina, LV dysfunction moderate, recent myocardial infarction, and emergency intervention. This final dataset represented the case-mix profile of 25114 patients included in the selected cohort and was used to generate the data for the simulation study.

#### Data generation

Using a bootstrap procedure, patients were resampled from the final dataset selected from the empirical data described above. As such, samples were constructed of a desired size containing patients with realistic case-mix profiles. For each bootstrap sample, the eight case-mix variables (*Z*1,…,*Z*8) were included as covariates in a multinomial logistic regression model to determine each patients probability of assignment to each provider:

1 $$  \pi_{k} = \frac{e^{\alpha_{k}+\beta_{k1}Z1+...+\beta_{k8}Z8}}{\sum\limits_{j}^{K} e^{\alpha_{j}+\beta_{j1}Z1+...+\beta_{j8}Z8}},  $$

where *k* represents a provider with *k*={1,…,*K*}, *α*_*k*_ is the provider-specific intercept and *β*_*k*1_,…,*β*_*k*8_ are the provider-specific coefficients for each case-mix variable. These coefficients were set equal within each provider (*β*_*k*1_=…=*β*_*k*8_), yet differed between providers, with coefficient values drawn from a uniform distribution between 0 and 1. The coefficients of one provider, which acted as reference, were all set to 0.

Patients were assigned a provider based on the probabilities calculated in Eq. 1. To ensure a fixed number of patients per provider as determined in each scenario, patients were continually resampled until each provider (*k*) had its required volume (*n*_*k*_) of patients. The amount of patients in the final sample (*N*) was dependent on the number of providers (*K*) and the volumes of the providers (*n*_*k*_), which varied over the scenarios described in section *Scenarios*.

Each patient’s value on the dichotomous outcome variable (*Y*) was generated using a random intercept logistic regression model:

2 $$  logit[\!p_{ik}]=\gamma_{00}+\alpha_{0k}+\beta'_{1}Z1_{ik}+...+\beta'_{8}Z8_{ik},  $$

where *p*_*ik*_ is the probability of mortality of the *i*th patient attending the *k*th provider, *γ*_00_ is the overall intercept, *α*_0*k*_ are the provider-specific random intercepts, and *Z*1_*ik*_,…,*Z*8_*ik*_ correspond to each patient’s scores on the case-mix variables. $\alpha _{0k}\sim \mathcal {N}\left (\mu,\sigma ^{2}\right)$, where *μ*=0 for normal providers and *μ*=±*H*∗*σ* for performance outliers that are either below or above average. *H* thus represents the amount of standard deviations by which the normal distribution is shifted when drawing the random intercepts of the *true* outlying providers. *σ* was set equal to 0.1942, corresponding to the standard deviation of the provider-specific intercepts found when fitting a random intercepts model on the full cohort of the dataset described in section *Data source*. When *H*=2 the mean of the random effects distributions of the outlying providers are then 0.3884 and -0.3884, corresponding to odds ratios of 1.475 and 0.678 respectively, keeping all else constant. Note that the overlap between the normal and outlier distributions is actually larger in practice, due to sampling variability. In a simple case, assuming an average incidence of the outcome of 10%, this distance is reduced to about 1.75∗*σ*.

The coefficients of the case-mix variables (*β*1′,…,*β*8′) corresponded to the odds ratios of the original EuroSCORE prediction model [53]. The average incidence of the outcome over all providers was fixed by manipulating the overall intercept (*γ*_00_) of the outcome model. In addition, each provider was required to have an incidence of the outcome of at least 1% to prevent separation and estimation problems when using the risk adjustment methods.

In this data generating mechanism, the case-mix variables acted as confounders of the provider-outcome relation. As no interaction terms were included in the model, the provider effects were assumed constant over the different levels of the case-mix variables. Given the use of a random intercepts model to generate the outcome, *LR*_*R*_ and the gPS methods were favored over *LR*_*F*_. Also note that both the gPS (Eq. 1) and outcome models (Eq. 2) were perfectly specified and contained the same relevant case-mix variables. While a strong assumption, this reduced the variability in performance over simulations and limited the complexity of the simulation study. As such, *LR*_*R*_ and *gPS*_*A*_ were expected to have comparable performance due to the similarity of the methods. Investigations into the consequences of model misspecification were outside the scope of the current study.

#### Scenarios

The parameters deemed relevant to manipulate are outlined below. Table 1 contains the parameter settings of the studied scenarios.

- The number of providers, *K*: 10, 20, 30, 40, or 50.

**Table 1:** Parameter Settings of Scenarios Studied Through Simulations

Scenario	*K*	*p*_··_	*P*(*out*)	*H*	*S*	*min*(*n*_*k*_)	*P*(*nmin*)
1	10-50	0.10	0.2	2	2	1000	0.5
2	50	0.03-0.20	0.2	2	2	1000	0.5
3	50	0.10	0.08-0.4	2	2	1000	0.5
4	50	0.10	0.2	1-4	2	1000	0.5
5	50	0.10	0.2	2	1-2	1000	0.5
6	50	0.10	0.2	2	2	500-1000	0.5
7	50	0.10	0.2	2	2	500	0.5-1

Shown are the Number of Providers (*K*), Average Mortality Rate Over Centers (*p*_··_), Percentage of True Outliers (*P*(*out*)), Factor by Which the Outlier Distributions Were Shifted (*H*), Amount of Sides That the Outliers Were Drawn From (*S*), Minimum Provider Volume (*min*(*n*_*k*_)), and the Probability of Outliers Being Small Providers (*P*(*nmin*))
- The average incidence of mortality, *p*_··_: 3, 10 or 20%.
- The percentage of true outliers, *P*(*out*): 8, 20 or 40%. This ensured an equal number of true outliers selected from both outlier distribution for each *K* studied.
- The amount of standard deviations the outlier random intercept distribution was shifted, *H*: 1, 2, 3, or 4.
- Outliers were either drawn from both outlier distributions (*S*=2) or only from the below-average performance distribution (*S*=1).
- Half of the providers were allocated either *min*(*n*_*k*_)=500 or *min*(*n*_*k*_)=1000 patients, while the other half were always of size *max*(*n*_*k*_)=1000.
- When *min*(*n*_*k*_)=500, on average either half, *P*(*nmin*)=0.5, or all, *P*(*nmin*)=1, of outlying providers had a sample size of 500. This allowed us to investigate the consequences of a potential correlation between provider volume and quality [17, 54, 55].

#### Statistical analysis

The risk adjustment methods introduced earlier, were applied on each of the generated datasets. In *LR*_*F*_ a logistic regression model only including the case-mix variables (*Z*1,…,*Z*8) was first fit to extract the overall intercept. Next, a second logistic regression model was fit without intercept including all *K* providers as dummy variables as well as *Z*1,…,*Z*8. Provider effects were classified as below or above average outliers if their 95% Wald confidence intervals did not include the overall intercept extracted from the first logistic regression model. In *LR*_*R*_ a random intercepts logistic regression model was fit including the *K* providers as random effects and *Z*1,…,*Z*8 as fixed effects. Providers of which the empirical Bayes effect estimate deviated more than two observed standard deviations from the overall intercept of the fitted model were classified as outliers.

For the four gPS methods applied to the generated data sets, outliers were classified in identical fashion as described for *LR*_*R*_. For *gPS*_*A*_ a random intercepts logistic regression model was fit including the *K* providers as random effects and *K*−1 gPSs as fixed effects. In *gPS*_*W*_, each patient was assigned a weight equal to the inverse of the gPS of the provider actually attended. A weighted random intercepts logistic regression was then performed as in *LR*_*R*_ with only the *K* providers included as random effects. *gPS*_*WT*_ was identical to *gPS*_*W*_, except that the highest 2% of weights were trimmed to the 98th percentile based on results from similar scenarios in [43]. The determination of the optimal trimming threshold was beyond the scope of this study. For *gPS*_*MWS*_ the gPSs for each provider were first stratified into *L*=5 strata, determined sufficient to remove over 90% of the selection bias [25, 56, 57]. Next the marginal mean weight (MMW) was calculated for each patient according to the formula described by Hong [44]:

3 $$ MMW= \frac{n_{s_{k}}*Pr(X=k)}{n_{X=k,s_{k}}},  $$

where $n_{s_{k}}$ is the number of patients in stratum *s* of provider *k*, *Pr*(*X*=*k*) is the proportion of patients assigned to provider *k* in the observed dataset and $n_{X=k,s_{k}}$ is the amount of patients in stratum *s*_*k*_ that actually attended provider *k*. The MMWs were then used to weight the sample as in *gPS*_*W*_ with the following analysis and outlier classification proceeding in an identical manner.

The logistic regression models in *LR*_*F*_ were fit using the function *glm* from the *stats* package, part of the R program [50]. The random intercept logistic regression models applied in all other methods (*LR*_*R*_, *gPS*_*A*_, *gPS*_*W*_, *gPS*_*WT*_, *gPS*_*MWS*_) were fit using the function *glmer* from the *lme4* package [58]. All models used in each method were properly specified, had the correct functional form and did not include interactions.

#### Classification performance

The classification accuracy of each risk adjustment method was evaluated by comparing the *observed* classification of each provider as normal or outlying with the *true* status, as determined when generating the data. While alternative methods are available to classify outliers, the approach presented above suffices to enable a fair comparison of the different risk adjustment methods. Traditional classification accuracy performance measures including sensitivity, specificity, positive predictive value (PPV), and negative predictive value (NPV) were computed for each generated data set and averaged over all simulations. In addition, 90th percentile confidence intervals were calculated for each of these performance measures. Finally, a measure of classification eagerness was considered by calculating the proportion of simulated datasets in which at least one outlier (not necessarily a true outlier) was observed.

## Results

**Fig. 1:**
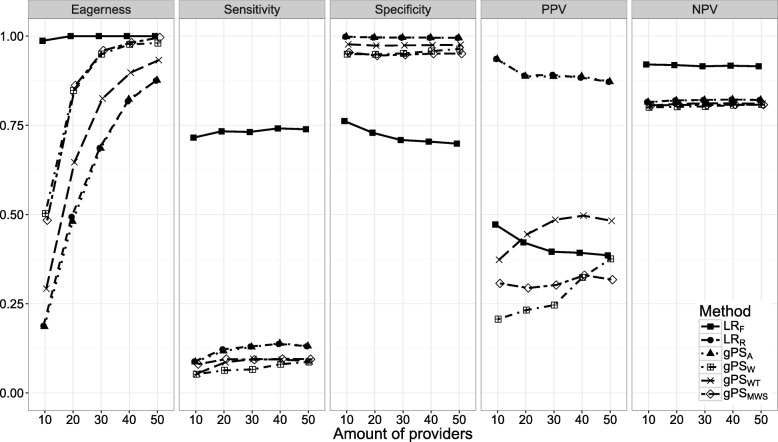
The eagerness, sensitivity, specificity, positive predictive value (PPV), and negative predictive value (NPV) for differing amounts of providers (*K*), when using different risk adjustment methods. All other parameters were fixed (see scenario 1 of Table 1)

**Fig. 2:**
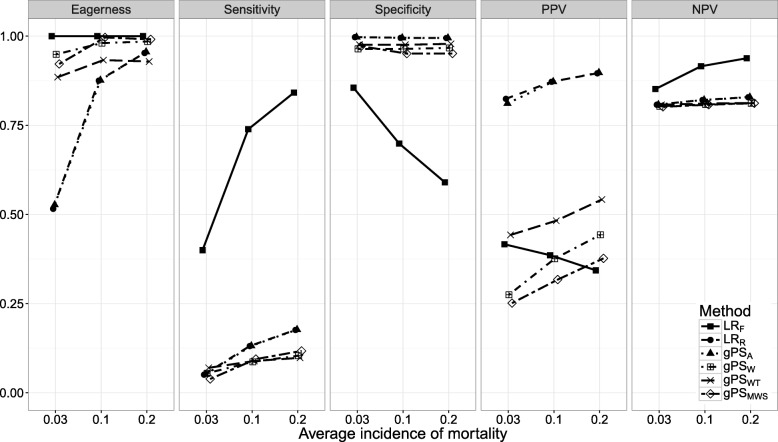
The eagerness, sensitivity, specificity, positive predictive value (PPV), and negative predictive value (NPV) for different average incidences of mortality (*p*_··_) when using different risk adjustment methods. All other parameters were fixed (see scenario 2 of Table 1)

**Fig. 3:**
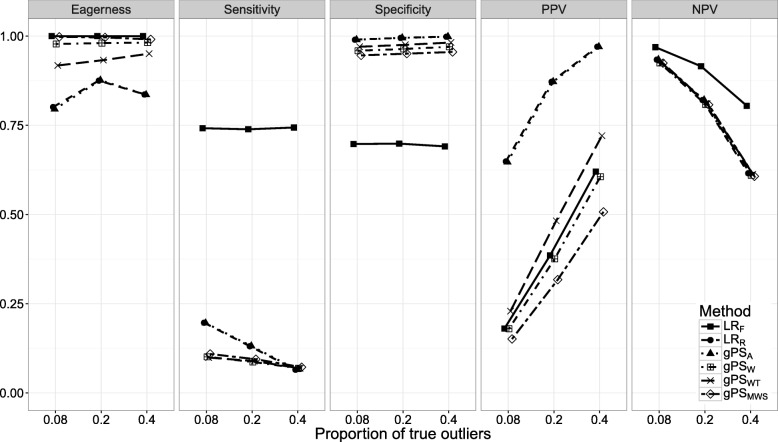
The eagerness, sensitivity, specificity, positive predictive value (PPV), and negative predictive value (NPV) for different proportions of true outliers (*P*(*out*)) when using different risk adjustment methods. All other parameters were fixed (see scenario 3 of Table 1)

**Fig. 4:**
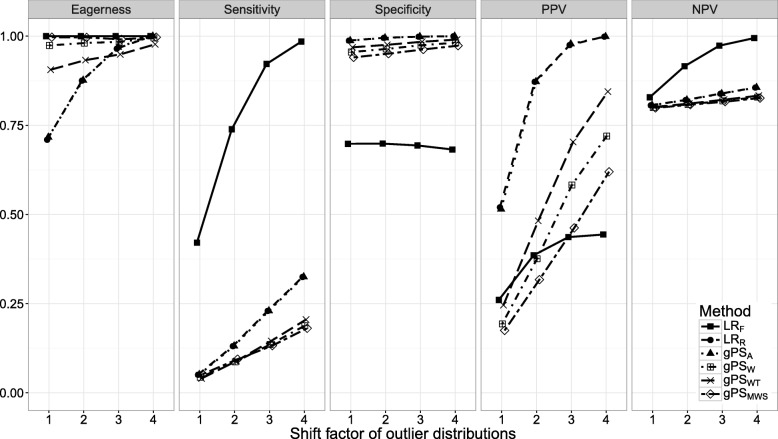
The eagerness, sensitivity, specificity, positive predictive value (PPV), and negative predictive value (NPV) for the factor by which the outlier distributions are shifted (*H*) when using different risk adjustment methods. All other parameters were fixed (see scenario 4 of Table 1)

**Fig. 5:**
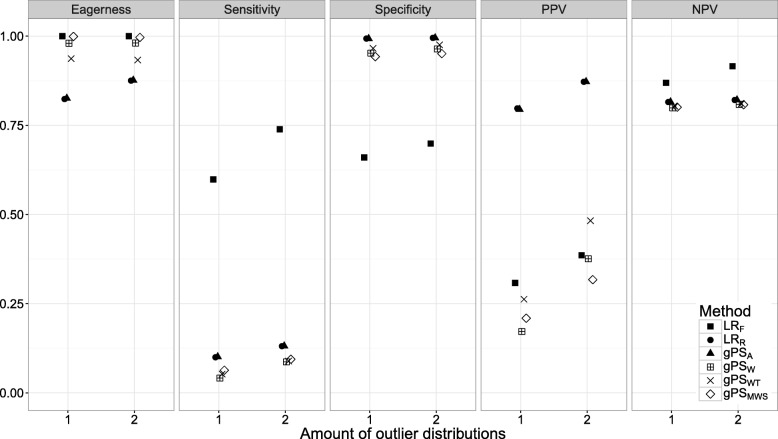
The eagerness, sensitivity, specificity, positive predictive value (PPV), and negative predictive value (NPV) for the amount of outlier distributions (*S*) when using different risk adjustment methods. All other parameters were fixed (see scenario 5 of Table 1)

**Fig. 6:**
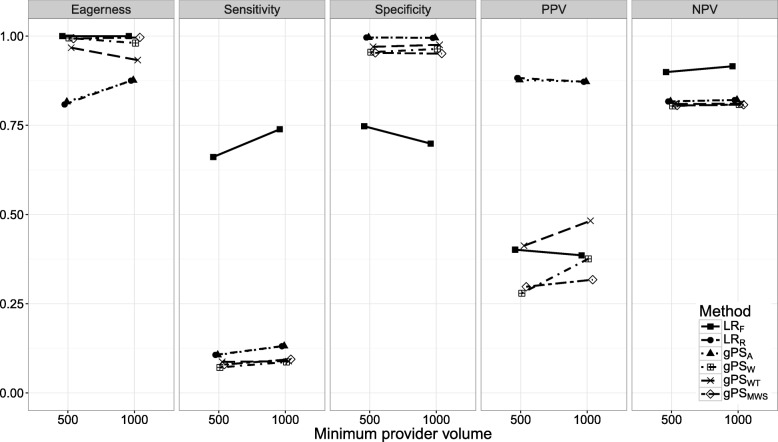
The eagerness, sensitivity, specificity, positive predictive value (PPV), and negative predictive value (NPV) for different minimum provider volumes, *min*(*n*_*k*_), when using different risk adjustment methods. All other parameters were fixed (see scenario 6 of Table 1)

**Fig. 7:**
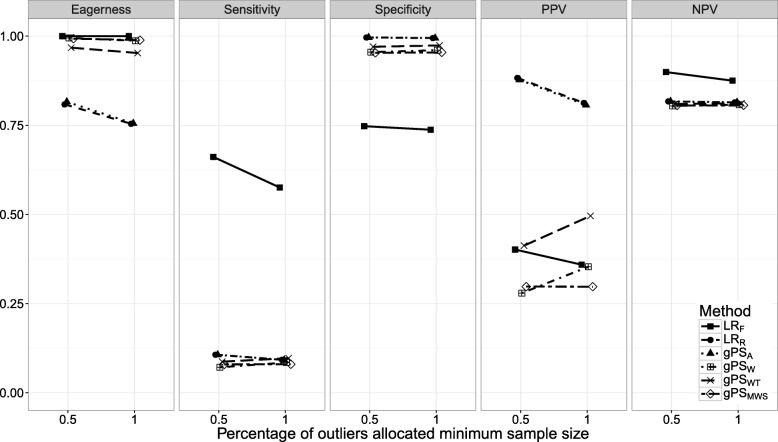
The eagerness, sensitivity, specificity, positive predictive value (PPV), and negative predictive value (NPV) for different percentages of outliers being allocated the minimum sample size, *P*(*nmin*), when using different risk adjustment methods. All other parameters were fixed (see scenario 7 of Table 1)

Figures 1, 2, 3, 4, 5, 6 and 7 show the classification performance of different risk adjustment methods for all studied scenarios (see Table 1). The 90th percentile confidence intervals over all bootstrap samples of these performance measures are displayed in Tables 2, 3, 4, 5, 6, 7 and 8 in the Appendix. Across all scenarios, the eagerness of *LR*_*F*_ surpassed that of the gPS methods and *LR*_*R*_. As these latter methods used random effects models to adjust for case-mix, conservativeness was to be expected. Of the gPS methods, *gPS*_*W*_ and *gPS*_*MWS*_ were most eager to identify outliers, while *gPS*_*A*_ was most conservative with a performance identical to *LR*_*R*_. *LR*_*F*_ consistently had a much higher sensitivity (∼ 75*%*) than the other methods (∼ 15*%*), of which *LR*_*R*_ and *gPS*_*A*_ scored several percentage points higher than their counterparts. gPS methods and *LR*_*R*_ had very high specificities (between 90 and 100%) across the board with *LR*_*F*_ coming in at 75%. As for the PPV, *LR*_*R*_ and *gPS*_*A*_ systematically scored best around 90%, with *LR*_*F*_,*gPS*_*W*_ and *gPS*_*MWS*_ performing worst with PPVs around 30%. With respect to the NPV, all gPS methods and *LR*_*R*_ had almost identical performance (∼ 80*%*). *LR*_*F*_ consistently scored about 10% higher.

*Scenario 1: number of providers*. Figure 1 shows the effect of *K* on classification performance. As expected, the eagerness of all methods quickly approach 100% for increasing *K*. Even though the sensitivity, specificity, and NPV of the gPS methods and *LR*_*R*_ seemed largely unaffected by *K*, *LR*_*R*_ and *gPS*_*A*_ had a slightly higher sensitivity compared to the other methods when *K* approached 50. While the PPV of *LR*_*R*_, *gPS*_*A*_, and *LR*_*F*_ decreased by about 8%, the PPV of *gPS*_*W*_ and *gPS*_*WT*_ increased by about 12 and 15% respectively. Meanwhile the sensitivity and NPV of *LR*_*F*_ was unaffected by *K*, while the specificity initially sloped downwards, before leveling off from *K*=30 onwards.

*Scenario 2: incidence of mortality*. In Fig. 2 the influence of *p*_··_ on classification performance was investigated. All methods approached an eagerness of 100% as *p*_··_ rose with *LR*_*R*_ and *gPS*_*A*_ increasing the most. When *p*_··_=0.03, the sensitivity of gPS methods and *LR*_*R*_ did not surpass 10% while that of *LR*_*F*_ dropped below 40%. As *p*_··_ increased, this rose by about 12% for *LR*_*R*_, *gPS*_*A*_ and *gPS*_*MWS*_, and over 45% for *LR*_*F*_. Only the specificity of *LR*_*F*_ was influenced by *p*_··_, dropping by about 25% as *p*_··_ increased. As for the PPV, all gPS methods and *LR*_*R*_ had a positive relationship with *p*_··_, while *LR*_*F*_ decreased as *p*_··_ rose. The NPV of all methods was mainly unaffected by *p*_··_; only *LR*_*F*_ dropped towards the level of the other methods when *p*_··_=0.03.

*Scenario 3: percentage of true outliers*. The influence of *P*(*out*) on classification performance is explored in Fig. 3. Increasing *P*(*out*) had little influence on the eagerness or specificity of all methods. Only the sensitivity of *LR*_*R*_ and *gPS*_*A*_ seemed to sharply decline towards the same level as the other gPS methods (7%) as *P*(*out*) increased. The PPV of all methods had a strong positive relationship with *P*(*out*), with *LR*_*F*_, *gPS*_*W*_ and *gPS*_*WT*_ rising by about 25%, and *LR*_*R*_ and *PS*_*A*_ rising by about 10%. The NPV of all methods decreased as *P*(*out*) increased. Especially the gPS methods and *LR*_*R*_ all decreased in identical fashion by over 20%. As both NPV and PPV are influenced by the prevalence (in our case the proportion of true outliers) these results were to be expected.

*Scenario 4: outlier distribution shift*. The relationship between *H* and classification performance was explored in Fig. 4. As expected, the eagerness of all methods increased towards 100% as *H* reached 4. The sensitivity of all methods was positively related to *H*, with *LR*_*F*_ increasing by more than 50% and *LR*_*R*_ and *gPS*_*A*_ by about 25%, about 10% more than the PS weighting methods. While the specificity remained unchanged, the PPV increased for all methods, with *gPS*_*WT*_ increasing most by about 50%. The PPV of *LR*_*F*_ leveled off after an increase of about 20%. The NPV of *LR*_*F*_ was the only one affected, increasing by about 20% as *H* approached 4.

*Scenarios 5 through 7*. The effect of *S*, *min*(*n*_*k*_), and *P*(*nmin*) on classification performance is shown in Figs. 5, 6 and 7. As expected, with performance outliers on both sides (*S*=2) all performance measures increased at least slightly for all methods. Including both small and large providers (*min*(*n*_*k*_)=500) had a small effect on classification performance. For *LR*_*F*_ the sensitivity and NPV increased slightly while the specificity and PPV decreased incrementally. Of the remaining methods, the PPV of *gPS*_*W*_ and *gPS*_*WT*_ and the eagerness of *gPS*_*A*_ and *LR*_*R*_ decreased slightly. When all outliers were allocated the minimum sample size (*P*(*nmin*)=1), the sensitivity, specificity and NPV of *LR*_*R*_ and the gPS methods was unchanged. For the PPV, *LR*_*R*_ and *gPS*_*A*_ slightly declined while *gPS*_*W*_ and *gPS*_*WT*_ slightly increased. All accuracy measures of *LR*_*F*_ slightly decreased as *P*(*nmin*) increased, with sensitivity dropping the most by about 10%.

## Discussion

In this study, the outlier classification performance of generalized propensity score (gPS) risk adjustment methods was compared to traditional regression-based methods when profiling multiple providers.Fixed effects logistic regression (*LR*_*F*_) consistently had the highest eagerness, sensitivity and negative predictive value (NPV), yet had a low specificity and positive predictive value (PPV). Of the random effects methods, gPS adjustment (*gPS*_*A*_) and random effects logistic regression (*LR*_*R*_) were the most conservative, yet performed equally well or better than all the remaining gPS methods for all classification accuracy measures across the studied scenarios. A decision on which of the studied methods to use should depend on the goal of the profiling exercise, taking into consideration the distinct differences between fixed and random effects risk adjustment methods outlined in section *Fixed and random effects logistic regression*.

While all gPS methods and *LR*_*R*_ used a random intercepts model in the analysis stage, *LR*_*F*_ solely included fixed effects. This was evident in the large performance differences between these methods and is in line with many published simulation studies examining fixed and random effects regression [6, 37, 39]. Also notable was the reactivity of *LR*_*F*_ to changes in most parameters as compared to the more stable random effects methods, for example with the sensitivity dropping sharply when outliers differed little from normal providers or the when the outcome was rare.

The sensitivity of all random effects methods was low across all scenarios. This was to be expected as the maximum achievable sensitivity was limited by the substantial overlap of the observed normal and outlier provider effect distributions. The degree of overlap was determined by the fixed standard deviation of the random effects distributions from which the effects were drawn, sampling variability and the distance between the normal and outlier distributions. When this distance (*H*) was increased the sensitivity quickly rose (see Fig. 4).

The overall identical performance of *gPS*_*A*_ and *LR*_*R*_ was to be expected given the inclusion of the same case-mix variables in both the gPS and outcome models. However, the inferior performance of the gPS weighting methods across all studied scenarios was surprising. Earlier findings suggested that gPS weighting (*gPS*_*W*_) outperformed gPS weighting with trimming (*gPS*_*WT*_) and had a performance on par with that of *gPS*_*A*_ and *LR*_*R*_ [28]. In the current study, trimming outlying weights had a positive effect on performance. Also unexpected was how gPS marginal mean weighting through stratification (*gPS*_*MWS*_) performed even worse in the majority of scenarios, disputing earlier claims of its superior performance [32, 44, 45]. A possible reason for this may be our omission of an essential step of the MMWS method, in which individuals that fall out of the common area of support are removed. This was done because for an increasing number of providers, effective sample sizes were reduced to 0. In addition, to ensure a fair and pragmatic comparison, the MMWS method was applied under similar circumstances as the other gPS methods where the assessment of balance across groups is often ignored because there is no consensus on how to do this properly when comparing multiple providers.

To explore the effect of many different parameters on classification performance, a simulation study was used. Due to the enormous amount of parameter combinations, a full factorial design was abandoned in favor of a univariate investigation of each parameter. Some parameter settings that might be seen in practice, such as provider volumes smaller than 500 patients, were omitted to prevent separation and convergence problems in addition to limiting the scope of the study. While scenarios were chosen to reflect realistic and sometimes extreme situations that may be encountered when profiling providers, more extensive investigation into the effect of the studied parameters and others may be necessary to judge the consistency of the results in all possible settings. Furthermore, several choices made when generating the data (such as the parameters of the provider effect distributions) will not reflect situations encountered in practice. Even so, it is not likely that this would affect the presented results. Strengths of the approach utilized in this paper were that the covariance structure of the case-mix variables was extracted from an empirical dataset and that associations between the case-mix variables and the outcome were taken from the original EuroSCORE model. Furthermore, drawing provider-specific random intercepts from three normal distribution of which only the mean differed was deemed theoretically realistic. This trimodal approach allowed the investigation of all cells of the confusion matrix and has been applied in similar simulation studies [6].

**Table 2:** The 90th percentile confidence intervals for all performance measure estimates of each method for Scenario 1 in Table 1 (corresponding to Fig. 1)

Measure	*K*	*L* *R*_*F*_	*L* *R*_*R*_	*g* *P* *S*_*A*_	*g* *P* *S*_*W*_	*g* *P* *S*_*WT*_	*g* *P* *S*_*MWS*_
Sensitivity	10	0.00, 1.00	0.00, 0.50	0.00, 0.50	0.00, 0.50	0.00, 0.50	0.00, 0.50
	20	0.25, 1.00	0.00, 0.25	0.00, 0.25	0.00, 0.25	0.00, 0.25	0.00, 0.25
	30	0.33, 1.00	0.00, 0.33	0.00, 0.33	0.00, 0.17	0.00, 0.33	0.00, 0.33
	40	0.50, 1.00	0.00, 0.25	0.00, 0.25	0.00, 0.25	0.00, 0.25	0.00, 0.25
	50	0.50, 0.90	0.00, 0.30	0.00, 0.30	0.00, 0.20	0.00, 0.20	0.00, 0.20
Specificity	10	0.50, 1.00	1.00, 1.00	1.00, 1.00	0.88, 1.00	0.88, 1.00	0.88, 1.00
	20	0.56, 0.88	0.94, 1.00	0.94, 1.00	0.88, 1.00	0.94, 1.00	0.88, 1.00
	30	0.54, 0.88	0.96, 1.00	0.96, 1.00	0.92, 1.00	0.92, 1.00	0.92, 1.00
	40	0.56, 0.84	0.97, 1.00	0.97, 1.00	0.91, 1.00	0.94, 1.00	0.91, 1.00
	50	0.57, 0.80	0.98, 1.00	0.98, 1.00	0.92, 1.00	0.95, 1.00	0.90, 1.00
PPV	10	0.00, 1.00	0.00, 1.00	0.00, 1.00	0.00, 1.00	0.00, 1.00	0.00, 1.00
	20	0.20, 0.67	0.00, 1.00	0.00, 1.00	0.00, 1.00	0.00, 1.00	0.00, 1.00
	30	0.25, 0.58	0.00, 1.00	0.00, 1.00	0.00, 1.00	0.00, 1.00	0.00, 1.00
	40	0.26, 0.55	0.00, 1.00	0.00, 1.00	0.00, 1.00	0.00, 1.00	0.00, 1.00
	50	0.27, 0.53	0.00, 1.00	0.00, 1.00	0.00, 1.00	0.00, 1.00	0.00, 1.00
NPV	10	0.75, 1.00	0.80, 0.89	0.80, 0.89	0.78, 0.89	0.78, 0.89	0.78, 0.89
	20	0.80, 1.00	0.80, 0.84	0.80, 0.84	0.78, 0.84	0.79, 0.84	0.78, 0.84
	30	0.82, 1.00	0.79, 0.86	0.79, 0.86	0.79, 0.83	0.79, 0.86	0.79, 0.85
	40	0.84, 1.00	0.79, 0.84	0.79, 0.84	0.78, 0.84	0.79, 0.84	0.78, 0.84
	50	0.84, 0.97	0.80, 0.85	0.80, 0.85	0.79, 0.83	0.79, 0.83	0.79, 0.83

*K* = number of providers; PPV = positive predictive value; NPV = negative predictive value

**Table 3:** The 90th percentile confidence intervals for all performance measure estimates of each method for Scenario 2 in Table 1 (corresponding to Fig. 2)

Measure	*p*_.._	*L* *R*_*F*_	*L* *R*_*R*_	*g* *P* *S*_*A*_	*g* *P* *S*_*W*_	*g* *P* *S*_*WT*_	*g* *P* *S*_*MWS*_
Sensitivity	0.03	0.20, 0.70	0.00, 0.20	0.00, 0.20	0.00, 0.20	0.00, 0.20	0.00, 0.10
	0.10	0.50, 0.90	0.00, 0.30	0.00, 0.30	0.00, 0.20	0.00, 0.20	0.00, 0.20
	0.20	0.68, 1.00	0.00, 0.30	0.00, 0.30	0.00, 0.20	0.00, 0.20	0.00, 0.30
Specificity	0.03	0.75, 0.95	0.97, 1.00	0.97, 1.00	0.92, 1.00	0.92, 1.00	0.95, 1.00
	0.10	0.57, 0.80	0.98, 1.00	0.98, 1.00	0.92, 1.00	0.95, 1.00	0.90, 1.00
	0.20	0.45, 0.72	0.97, 1.00	0.97, 1.00	0.92, 1.00	0.95, 1.00	0.90, 1.00
PPV	0.03	0.18, 0.67	0.00, 1.00	0.00, 1.00	0.00, 1.00	0.00, 1.00	0.00, 1.00
	0.10	0.27, 0.53	0.00, 1.00	0.00, 1.00	0.00, 1.00	0.00, 1.00	0.00, 1.00
	0.20	0.26, 0.43	0.50, 1.00	0.50, 1.00	0.00, 1.00	0.00, 1.00	0.00, 1.00
NPV	0.03	0.80, 0.92	0.80, 0.83	0.80, 0.83	0.79, 0.83	0.79, 0.83	0.79, 0.82
	0.10	0.84, 0.97	0.80, 0.85	0.80, 0.85	0.79, 0.83	0.79, 0.83	0.79, 0.83
	0.20	0.86, 1.00	0.80, 0.85	0.80, 0.85	0.79, 0.83	0.79, 0.83	0.79, 0.85

*p*_.._ = average mortality rate over providers; PPV = positive predictive value; NPV = negative predictive value

**Table 4:** The 90th percentile confidence intervals for all performance measure estimates of each method for Scenario 3 in Table 1 (corresponding to Fig. 3)

Measure	*P*(*out*)	*L* *R*_*F*_	*L* *R*_*R*_	*g* *P* *S*_*A*_	*g* *P* *S*_*W*_	*g* *P* *S*_*WT*_	*g* *P* *S*_*MWS*_
Sensitivity	0.03	0.20, 0.70	0.00, 0.20	0.00, 0.20	0.00, 0.20	0.00, 0.20	0.00, 0.10
	0.10	0.50, 0.90	0.00, 0.30	0.00, 0.30	0.00, 0.20	0.00, 0.20	0.00, 0.20
	0.20	0.68, 1.00	0.00, 0.30	0.00, 0.30	0.00, 0.20	0.00, 0.20	0.00, 0.30
Specificity	0.03	0.75, 0.95	0.97, 1.00	0.97, 1.00	0.92, 1.00	0.92, 1.00	0.95, 1.00
	0.10	0.57, 0.80	0.98, 1.00	0.98, 1.00	0.92, 1.00	0.95, 1.00	0.90, 1.00
	0.20	0.45, 0.72	0.97, 1.00	0.97, 1.00	0.92, 1.00	0.95, 1.00	0.90, 1.00
PPV	0.03	0.18, 0.67	0.00, 1.00	0.00, 1.00	0.00, 1.00	0.00, 1.00	0.00, 1.00
	0.10	0.27, 0.53	0.00, 1.00	0.00, 1.00	0.00, 1.00	0.00, 1.00	0.00, 1.00
	0.20	0.26, 0.43	0.50, 1.00	0.50, 1.00	0.00, 1.00	0.00, 1.00	0.00, 1.00
NPV	0.03	0.80, 0.92	0.80, 0.83	0.80, 0.83	0.79, 0.83	0.79, 0.83	0.79, 0.82
	0.10	0.84, 0.97	0.80, 0.85	0.80, 0.85	0.79, 0.83	0.79, 0.83	0.79, 0.83
	0.20	0.86, 1.00	0.80, 0.85	0.80, 0.85	0.79, 0.83	0.79, 0.83	0.79, 0.85

*P*(*out*) = percentage of true outliers; PPV = positive predictive value; NPV = negative predictive value

To limit the complexity and scope of the simulation study, several important features of the risk adjustment process were disregarded. When applying PS methods it is essential to assess the overlap of the estimated PS distributions prior to fitting the outcome model. However, this step is often omitted in practice as there is no consensus on how to assess balance on multiple case-mix variables when considering more than three providers. Disregarding it in our simulation study allowed us to evaluate all methods including their drawbacks. Furthermore, all PS and outcome models were assumed properly specified with no unmeasured confounding by including all the case-mix variables used for data generation in the analysis phase. While the authors acknowledge that performance of the risk adjustment methods may differ in more realistic situations, the results from this controlled simulation study may act as reference for essential further studies into the effect of misspecification and unobserved confounding on classification performance. Several authors have already recently commented on the potential effects of misspecification in comparable, yet simpler situations [59, 60]. Lastly, it is important to stress that the data was generated under the assumption of a random intercepts model, and thus inherently favored the random effects methods. Further simulation studies may be performed to investigate the effect of using different data generating mechanisms on the performance of the considered risk adjustment methods.

## Conclusions

This study has demonstrated that of the gPS methods studied, only gPS case-mix adjustment can be considered as a viable alternative to random effects logistic regression when profiling multiple providers in different scenarios. The former method may be preferred as it allows the assessment of balance across providers prior to fitting the outcome model. Additionally, the many different scenarios investigated can give guidance on the classification performance that may be expected when dealing with different provider profiling exercises.

## Appendix

### Confidence interval tables

The following 7 tables show the 90th percentile confidence intervals of the performance measures assessed for each method within each scenario. Note that the number of true outliers in the studied scenarios depended on the amount of providers (*K*) and the percentage of true outliers (*P*(*out*)). As a result performance measures such as the sensitivity could only take on a limited number of values within each sample. In addition, a difference of one in the number of outliers observed would also lead to a relatively large change in the studied performance measures.

**Table 5:** The 90th percentile confidence intervals for all performance measure estimates of each method for Scenario 4 in Table 1 (corresponding to Fig. 4)

Measure	*H*	*L* *R*_*F*_	*L* *R*_*R*_	*g* *P* *S*_*A*_	*g* *P* *S*_*W*_	*g* *P* *S*_*WT*_	*g* *P* *S*_*MWS*_
Sensitivity	1	0.20, 0.70	0.00, 0.20	0.00, 0.20	0.00, 0.20	0.00, 0.10	0.00, 0.20
	2	0.50, 0.90	0.00, 0.30	0.00, 0.30	0.00, 0.20	0.00, 0.20	0.00, 0.20
	3	0.80, 1.00	0.10, 0.40	0.10, 0.40	0.00, 0.30	0.00, 0.30	0.00, 0.30
	4	0.90, 1.00	0.20, 0.50	0.20, 0.50	0.00, 0.30	0.00, 0.40	0.00, 0.40
Specificity	1	0.57, 0.82	0.95, 1.00	0.95, 1.00	0.92, 1.00	0.92, 1.00	0.90, 0.97
	2	0.57, 0.80	0.98, 1.00	0.98, 1.00	0.92, 1.00	0.95, 1.00	0.90, 1.00
	3	0.57, 0.82	0.97, 1.00	0.97, 1.00	0.95, 1.00	0.95, 1.00	0.92, 1.00
	4	0.55, 0.80	1.00, 1.00	1.00, 1.00	0.95, 1.00	0.97, 1.00	0.95, 1.00
PPV	1	0.11, 0.41	0.00, 1.00	0.00, 1.00	0.00, 1.00	0.00, 1.00	0.00, 0.50
	2	0.27, 0.53	0.00, 1.00	0.00, 1.00	0.00, 1.00	0.00, 1.00	0.00, 1.00
	3	0.33, 0.56	0.75, 1.00	0.75, 1.00	0.00, 1.00	0.00, 1.00	0.00, 1.00
	4	0.36, 0.56	1.00, 1.00	1.00, 1.00	0.00, 1.00	0.33, 1.00	0.00, 1.00
NPV	1	0.76, 0.90	0.79, 0.83	0.79, 0.83	0.79, 0.83	0.79, 0.82	0.78, 0.83
	2	0.84, 0.97	0.80, 0.85	0.80, 0.85	0.79, 0.83	0.79, 0.83	0.79, 0.83
	3	0.92, 1.00	0.81, 0.87	0.82, 0.87	0.79, 0.85	0.80, 0.85	0.79, 0.85
	4	0.96, 1.00	0.83, 0.89	0.83, 0.89	0.80, 0.85	0.80, 0.87	0.79, 0.87

*H* = factor by which the outlier distributions were shifted; PPV = positive predictive value; NPV = negative predictive value

**Table 6:** The 90th percentile confidence intervals for all performance measure estimates of each method for Scenario 5 in Table 1 (corresponding to Fig. 5)

Measure	*S*	*L* *R*_*F*_	*L* *R*_*R*_	*g* *P* *S*_*A*_	*g* *P* *S*_*W*_	*g* *P* *S*_*WT*_	*g* *P* *S*_*MWS*_
Sensitivity	1	0.40, 0.80	0.00, 0.20	0.00, 0.20	0.00, 0.10	0.00, 0.20	0.00, 0.20
	2	0.50, 0.90	0.00, 0.30	0.00, 0.30	0.00, 0.20	0.00, 0.20	0.00, 0.20
Specificity	1	0.55, 0.78	0.97, 1.00	0.97, 1.00	0.92, 1.00	0.92, 1.00	0.90, 0.97
	2	0.57, 0.80	0.98, 1.00	0.98, 1.00	0.92, 1.00	0.95, 1.00	0.90, 1.00
PPV	1	0.20, 0.41	0.00, 1.00	0.00, 1.00	0.00, 0.67	0.00, 1.00	0.00, 0.67
	2	0.27, 0.53	0.00, 1.00	0.00, 1.00	0.00, 1.00	0.00, 1.00	0.00, 1.00
NPV	1	0.80, 0.93	0.80, 0.83	0.80, 0.83	0.79, 0.82	0.79, 0.83	0.78, 0.83
	2	0.84, 0.97	0.80, 0.85	0.80, 0.85	0.79, 0.83	0.79, 0.83	0.79, 0.83

*S* = amount of sides that the outliers were drawn from; PPV = positive predictive value; NPV = negative predictive value

**Table 7:** The 90th percentile confidence intervals for all performance measure estimates of each method for Scenario 6 in Table 1 (corresponding to Fig. 6)

Measure	*min*(*n*_*k*_)	*L* *R*_*F*_	*L* *R*_*R*_	*g* *P* *S*_*A*_	*g* *P* *S*_*W*_	*g* *P* *S*_*WT*_	*g* *P* *S*_*MWS*_
Sensitivity	500	0.40, 0.90	0.00, 0.20	0.00, 0.20	0.00, 0.20	0.00, 0.20	0.00, 0.20
	1000	0.50, 0.90	0.00, 0.30	0.00, 0.30	0.00, 0.20	0.00, 0.20	0.00, 0.20
Specificity	500	0.62, 0.85	0.97, 1.00	0.97, 1.00	0.92, 1.00	0.92, 1.00	0.90, 1.00
	1000	0.57, 0.80	0.98, 1.00	0.98, 1.00	0.92, 1.00	0.95, 1.00	0.90, 1.00
PPV	500	0.26, 0.56	0.00, 1.00	0.00, 1.00	0.00, 1.00	0.00, 1.00	0.00, 1.00
	1000	0.27, 0.53	0.00, 1.00	0.00, 1.00	0.00, 1.00	0.00, 1.00	0.00, 1.00
NPV	500	0.83, 0.97	0.80, 0.83	0.80, 0.83	0.79, 0.83	0.79, 0.83	0.79, 0.83
	1000	0.84, 0.97	0.80, 0.85	0.80, 0.85	0.79, 0.83	0.79, 0.83	0.79, 0.83

*min*(*n*_*k*_) = minimum provider volume; PPV = positive predictive value; NPV = negative predictive value

**Table 8:** The 90th percentile confidence intervals for all performance measure estimates of each method for Scenario 7 in Table 1 (corresponding to Fig. 7)

Measure	*P*(*nmin*)	*L* *R*_*F*_	*L* *R*_*R*_	*g* *P* *S*_*A*_	*g* *P* *S*_*W*_	*g* *P* *S*_*WT*_	*g* *P* *S*_*MWS*_
Sensitivity	0.5	0.40, 0.90	0.00, 0.20	0.00, 0.20	0.00, 0.20	0.00, 0.20	0.00, 0.20
	1	0.30, 0.80	0.00, 0.20	0.00, 0.20	0.00, 0.20	0.00, 0.20	0.00, 0.20
Specificity	0.5	0.62, 0.85	0.97, 1.00	0.97, 1.00	0.92, 1.00	0.92, 1.00	0.90, 1.00
	1	0.62, 0.85	0.98, 1.00	0.98, 1.00	0.92, 1.00	0.92, 1.00	0.92, 1.00
PPV	0.5	0.26, 0.56	0.00, 1.00	0.00, 1.00	0.00, 1.00	0.00, 1.00	0.00, 1.00
	1	0.21, 0.50	0.00, 1.00	0.00, 1.00	0.00, 1.00	0.00, 1.00	0.00, 1.00
NPV	0.5	0.83, 0.97	0.80, 0.83	0.80, 0.83	0.79, 0.83	0.79, 0.83	0.79, 0.83
	1	0.81, 0.94	0.80, 0.83	0.80, 0.83	0.79, 0.83	0.79, 0.83	0.79, 0.83

*P*(*nmin*) = probability of outliers being small providers; PPV = positive predictive value; NPV = negative predictive value

## Abbreviations

CABG: Coronary artery bypass grafting
PS: Propensity score
gPS: Generalized propensity score
*L**R*_*F*_: fixed effects logistic regression
*L**R*_*R*_: random effects logistic regression
*g**P**S*_*A*_: gPS case-mix adjustment
*g**P**S*_*W*_: gPS inverse probability weighting
*g**P**S*_*WT*_: gPS inverse probability weighting with trimming
*g**P**S*_*MWS*_: gPS marginal mean weighting through stratification
mmw: Marginal mean weight
ppv: Positive predictive value
npv: Negative predictive value
